# Supplementary material for: Autobehaver: An AI-Based Pipeline for Animal Behavior Analysis
Source: bioRxiv. 2026 May 15:2026.05.12.724596. Preprint. [Version 1] doi: 10.64898/2026.05.12.724596 (PMC13192891; doi:10.64898/2026.05.12.724596)
Supplement: 1 [file NIHPP2026.05.12.724596V1-supplement-1.pdf]

bioRxiv preprint doi: https://doi.org/10.64898/2026.05.12.724596; this version posted May 15, 2026. The copyright holder for this preprint (which was not certified by peer review) is the author/funder. This article is a US Government work. It is not subject to copyright under 17 USC 105 and is also made available for use under a CC0 license.

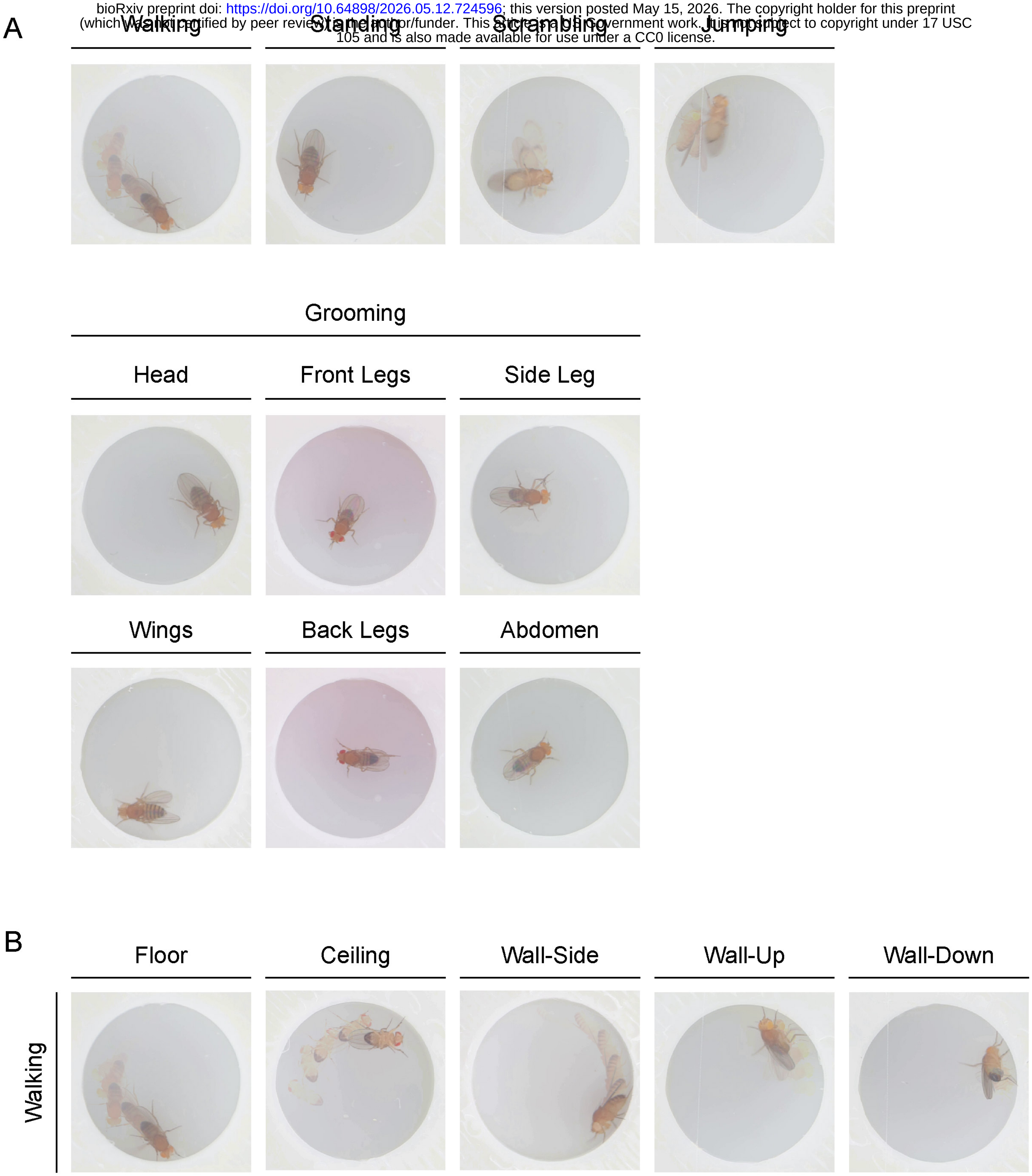

Supplemental Figure 1 - Behaviors and Orientations

bioRxiv preprint doi: https://doi.org/10.64898/2026.05.12.724596; this version posted May 15, 2026. The copyright holder for this preprint (which was not certified by peer review) is the author/funder. This article is a US Government work. It is not subject to copyright under 17 USC 105 and is also made available for use under a CC0 license.

Supplemental Figure 1: Behaviors and Orientations

The 10 annotated Behaviors (A) included Walking, Standing, Scrambling, Jumping, and six types of Grooming: Head, Front Legs, Side Leg, Wings, Back Legs, and Abdomen. The five annotated Orientations (B) are shown in combination with Walking Behavior. Dynamic poses are shown in stroboscopic view to depict movement.

bioRxiv preprint doi: https://doi.org/10.64898/2026.05.12.724596; this version posted May 15, 2026. The copyright holder for this preprint (which was not certified by peer review) is the author/funder. This article is a US Government work. It is not subject to copyright under 17 USC 105 and is also made available for use under a CC0 license.

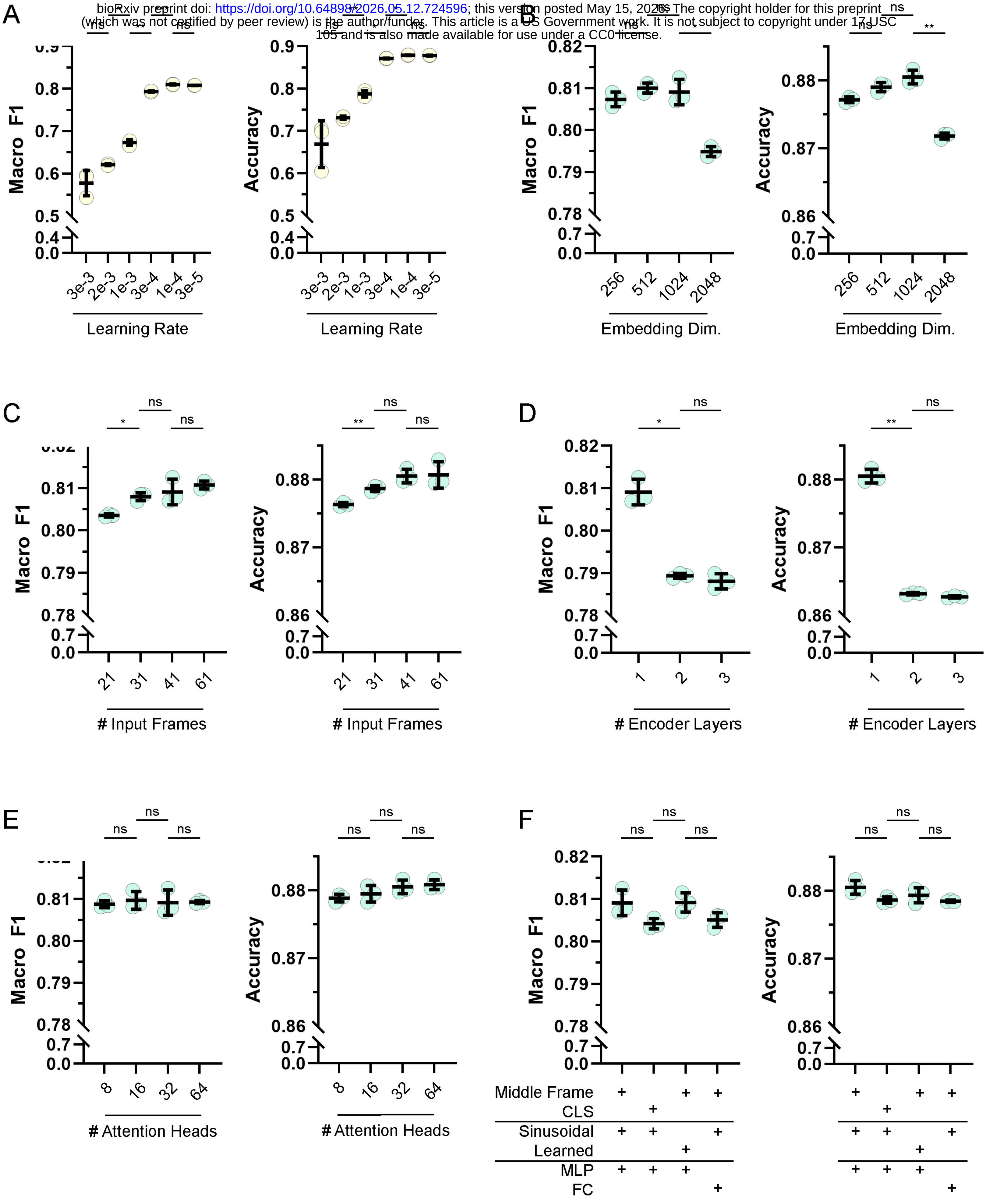

Figure S2 - Hyperparameter and Architecture Tuning Results

bioRxiv preprint doi: https://doi.org/10.64898/2026.05.12.724596; this version posted May 15, 2026. The copyright holder for this preprint (which was not certified by peer review) is the author/funder. This article is a US Government work. It is not subject to copyright under 17 USC 105 and is also made available for use under a CC0 license.

Supplemental Figure 2: Hyperparameter and Architecture Tuning Results

Architecture tuning experiments were performed using a dataset containing all features, a predefined training-validation split, predefined random seeds, and a common set of architecture parameters with a single change for each experiment. Results are reported as Validation Macro F1 (A, C, E, G, I, K) and Validation Accuracy (B, D, F, H, J, L) for three replicates, with column labels indicating the altered parameter(s). First, base learning rates were tested (A, B) using common parameters of 41 input frames, one encoder layer, an embedding dimension of 512, 32 attention heads, sinusoidal positional encodings, a middle-frame representation, multi-layer perceptron (MLP) prediction heads, and a 300-epoch cosine decay schedule with a final learning rate of 0.000001. Second, embedding dimensions (C, D) were tested using the same common parameters and base learning rate 0.0001. Third, the number of input frames (E, F), encoder layers (G, H), and attention heads (I, J) were tested using the same common parameters, except embedding dimension which was set to 1024. We also tested CLS token instead of middle frame token representation, learned instead of sinusoidal positional encodings, and fully connected (FC) instead of MLP prediction heads (K, L). Statistical comparisons were made using Welch's ANOVA with Dunnett's T3 post-hoc test for all-to-all pairwise comparisons. *** $p < 0.001$; ** $p < 0.01$; * $p < 0.05$; ns, not significant. Only selected statistical results are shown for simplicity.

bioRxiv preprint doi: [illegible]; this version posted [illegible]. The copyright holder for this preprint
(which was not certified by peer review) is the author/funder. This article is a US Government work. It is not subject to copyright under 17 USC 105 and is also made available for use under a CC0 license.

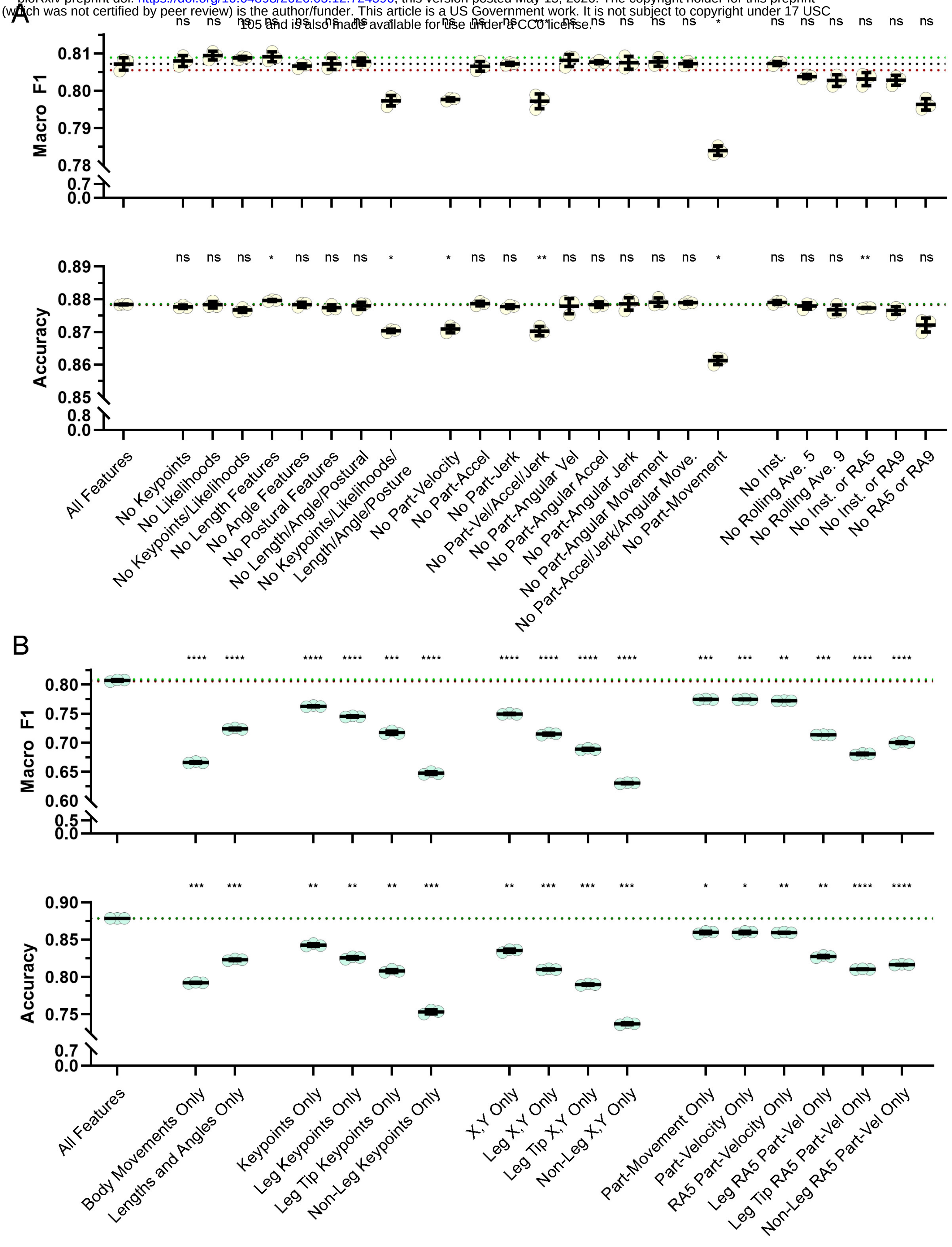

Figure S3 - Dataset Ablation Experiments

bioRxiv preprint doi: https://doi.org/10.64898/2026.05.12.724596; this version posted May 15, 2026. The copyright holder for this preprint (which was not certified by peer review) is the author/funder. This article is a US Government work. It is not subject to copyright under 17 USC 105 and is also made available for use under a CC0 license.

Supplemental Figure 3: Dataset Ablation Experiments

Dataset ablation experiments were performed using a predefined training-validation split, predefined random seeds, and a common set of architecture parameters: 41 input frames, one encoder layer, an embedding dimension of 1024, 32 attention heads, sinusoidal positional encodings, middle-frame representation, multi-layer perceptron prediction heads, and a 300-epoch cosine decay schedule with base learning rate 0.0001 and final learning rate of 0.000001. Results are reported as both validation Macro F1 (top) and Accuracy (bottom) for each of three replicates, with the mean and upper and lower standard deviation bounds of the "All Features" model shown as black, green, and red dotted lines, respectively. Experiments were performed by either dropping feature subsets (A) or by training on only one feature subset (B). Statistical comparisons were made using Welch's ANOVA with Dunnett's T3 post-hoc test for pairwise comparisons against "All Features". **** $p < 0.0001$; *** $p < 0.001$; ** $p < 0.01$; * $p < 0.05$; ns, not significant.

bioRxiv preprint doi: https://doi.org/10.64898/2026.05.12.724596; this version posted May 15, 2026. The copyright holder for this preprint (which was not certified by peer review) is the author/funder. This article is a US Government work. It is not subject to copyright under 17 USC 105 and is also made available for use under a CC0 license.

A

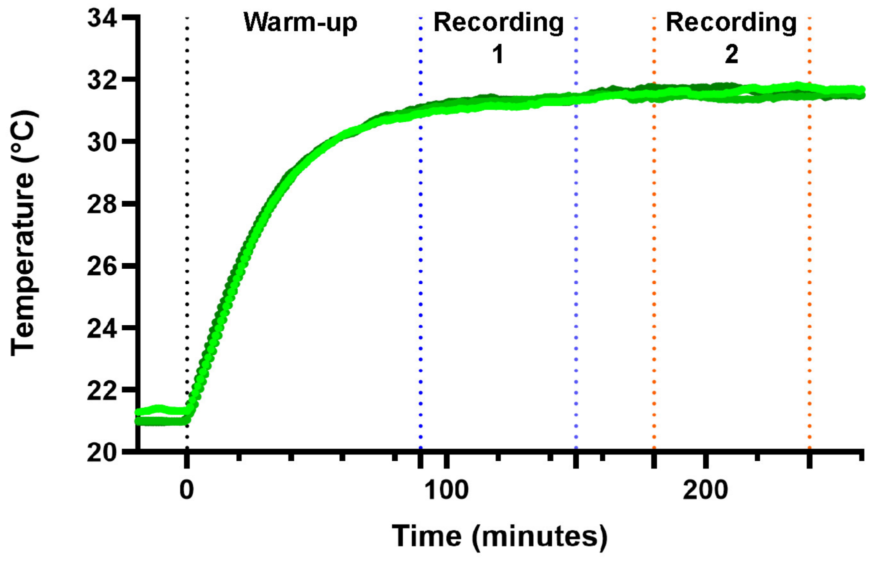

Figure S4 - Temperature During Recording

bioRxiv preprint doi: https://doi.org/10.64898/2026.05.12.724596; this version posted May 15, 2026. The copyright holder for this preprint (which was not certified by peer review) is the author/funder. This article is a US Government work. It is not subject to copyright under 17 USC 105 and is also made available for use under a CC0 license.

Supplemental Figure 4: Temperature During Recording

Temperature recordings from inside the imaging box showed temperature eventually plateaued at 31.8°C (exponential one-phase association regression, R^2 0.965). The first recording could be initiated as early as 90 minutes, at which point temperature rose from 31°C to 31.4°C over the recording period. The second recording could be initiated as early as 180 minutes, when temperature was stable at 31.6°C.

bioRxiv preprint doi: https://doi.org/10.64898/2026.05.12.724596; this version posted May 15, 2026. The copyright holder for this preprint (which was not certified by peer review) is the author/funder. This article is a US Government work. It is not subject to copyright under 17 USC 105 and is also made available for use under a CC0 license.

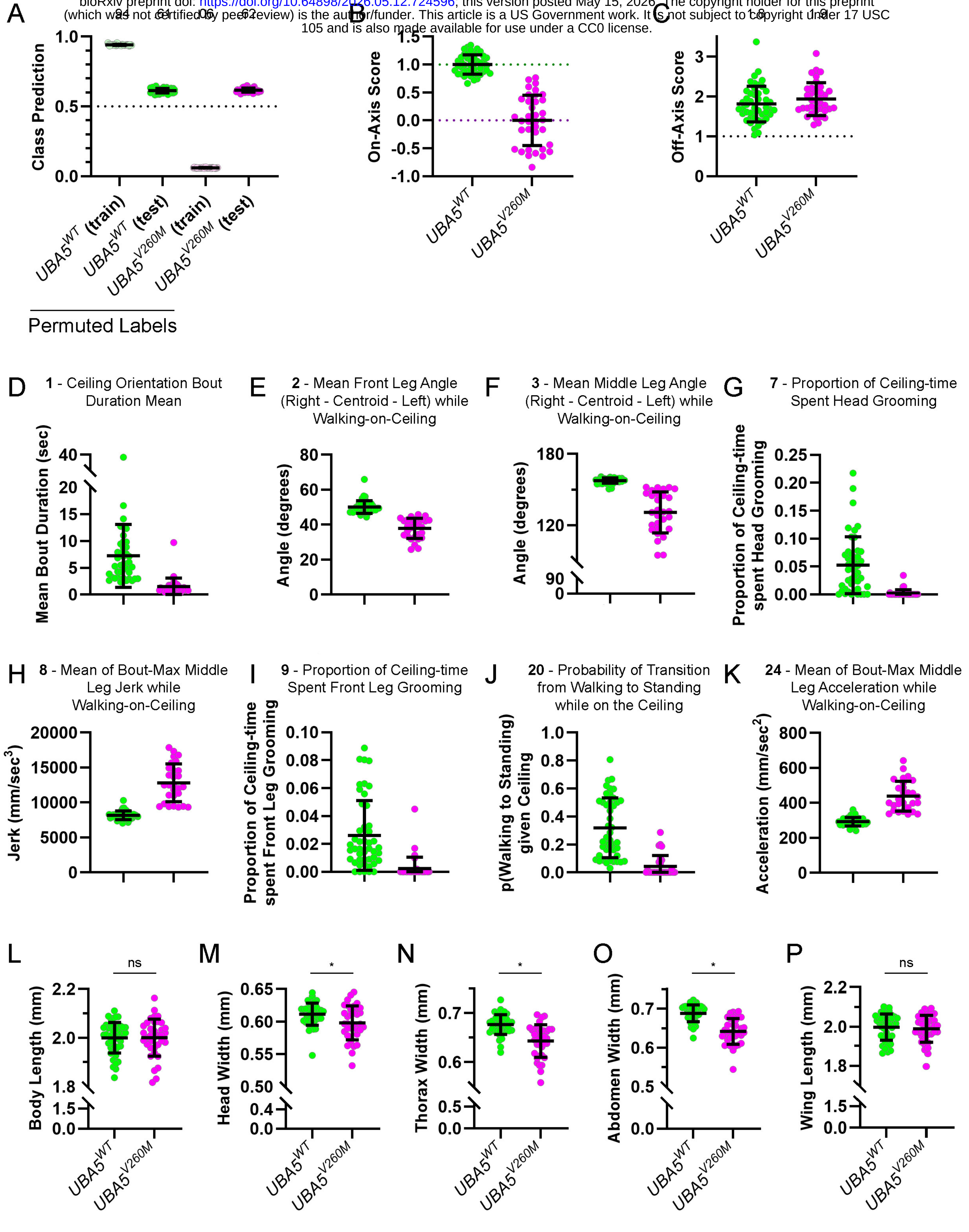

Figure S5 - *UBA5* Supporting Information

bioRxiv preprint doi: https://doi.org/10.64898/2026.05.12.724596; this version posted May 15, 2026. The copyright holder for this preprint (which was not certified by peer review) is the author/funder. This article is a US Government work. It is not subject to copyright under 17 USC 105 and is also made available for use under a CC0 license.

Supplemental Figure 5: *UBA5* Supporting Information

Training a classifier ensemble on *UBA5* individuals with permuted labels (A) resulted in no discriminative power (average class prediction for non-reference individuals = 0.62). The full behavioral feature matrix was projected into principal component space to define an axis separating the centroids of $UBA5^{WT}$ and $UBA5^{V260M}$ individuals, resulting in on-axis (B) and off-axis (C) components for each individual, both reported in the same unitless scale. On-axis values indicate each fly's position along the line connecting the average positions of the two reference groups in PCA space; off-axis values indicate each fly's distance from that line. Raw feature plots for selected top-ranked Ceiling Orientation-related features are shown, with the number preceding each feature name indicating its rank in the global-importance list: ceiling bout duration mean (D); mean angle formed between the front right tarsus, centroid, and front left tarsus while Walking on the Ceiling (E); mean angle formed between the middle right tarsus, centroid, and middle left tarsus while Walking on the Ceiling (F); proportion of total Ceiling time spent Head Grooming (G); mean of bout-specific maximum middle leg jerk while Walking on the Ceiling (H); proportion of total Ceiling time spent Front Leg Grooming (I); probability of transitioning from Walking to Standing while on the Ceiling (J); and mean of bout-specific maximum middle leg acceleration while Walking on the Ceiling (K). Standard measurements of $UBA5^{WT}$ and $UBA5^{V260M}$ individuals are shown for body length (L), head width (M), thorax width (N), abdomen width (O), and wing length (P). Statistical significance was determined by Welch's t-test. * $p < 0.05$; ns, not significant.

bioRxiv preprint doi: https://doi.org/10.64898/2026.05.12.724596; this version posted May 15, 2026. The copyright holder for this preprint
(which was not certified by peer review) is the author/funder. This article is a US Government work. It is not subject to copyright under 17 USC
105 and is also made available for use under a CC0 license.

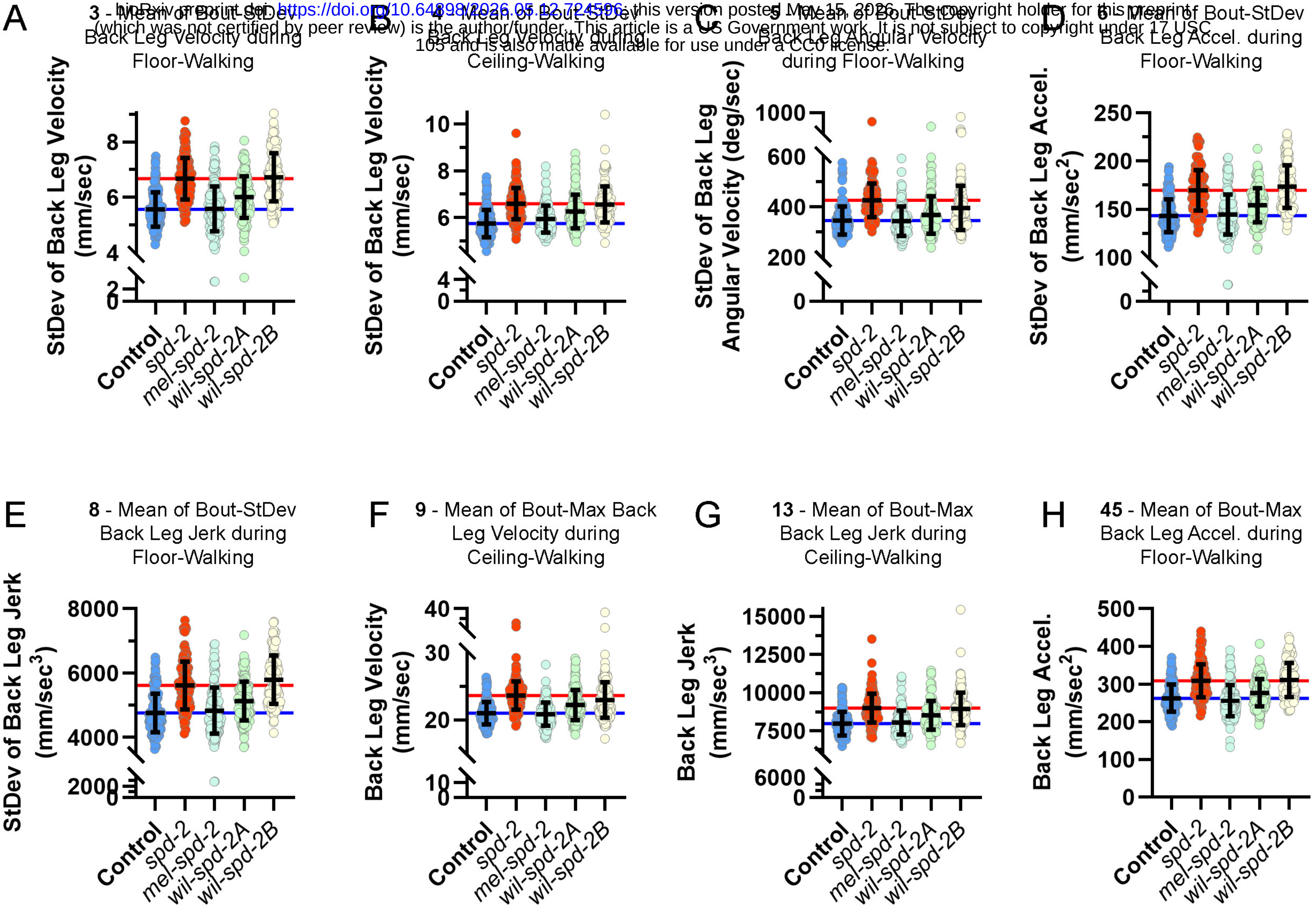

Figure S6 - *Spd-2* Supporting Information

bioRxiv preprint doi: https://doi.org/10.64898/2026.05.12.724596; this version posted May 15, 2026. The copyright holder for this preprint (which was not certified by peer review) is the author/funder. This article is a US Government work. It is not subject to copyright under 17 USC 105 and is also made available for use under a CC0 license.

Supplemental Figure 6: *Spd-2* Supporting Information

Raw feature plots for selected top-ranked features from the feature group describing back leg kinematics during Walking bouts (A-H). The number preceding each feature name indicates its rank in the global importance-ranked list. Horizontal colored lines indicate the mean values for control (blue) and *spd-2* (red).

bioRxiv preprint doi: https://doi.org/10.64898/2026.05.12.724596; this version posted May 15, 2026. The copyright holder for this preprint (which was not certified by peer review) is the author/funder. This article is a US Government work. It is not subject to copyright under 17 USC 105 and is also made available for use under a CC0 license.

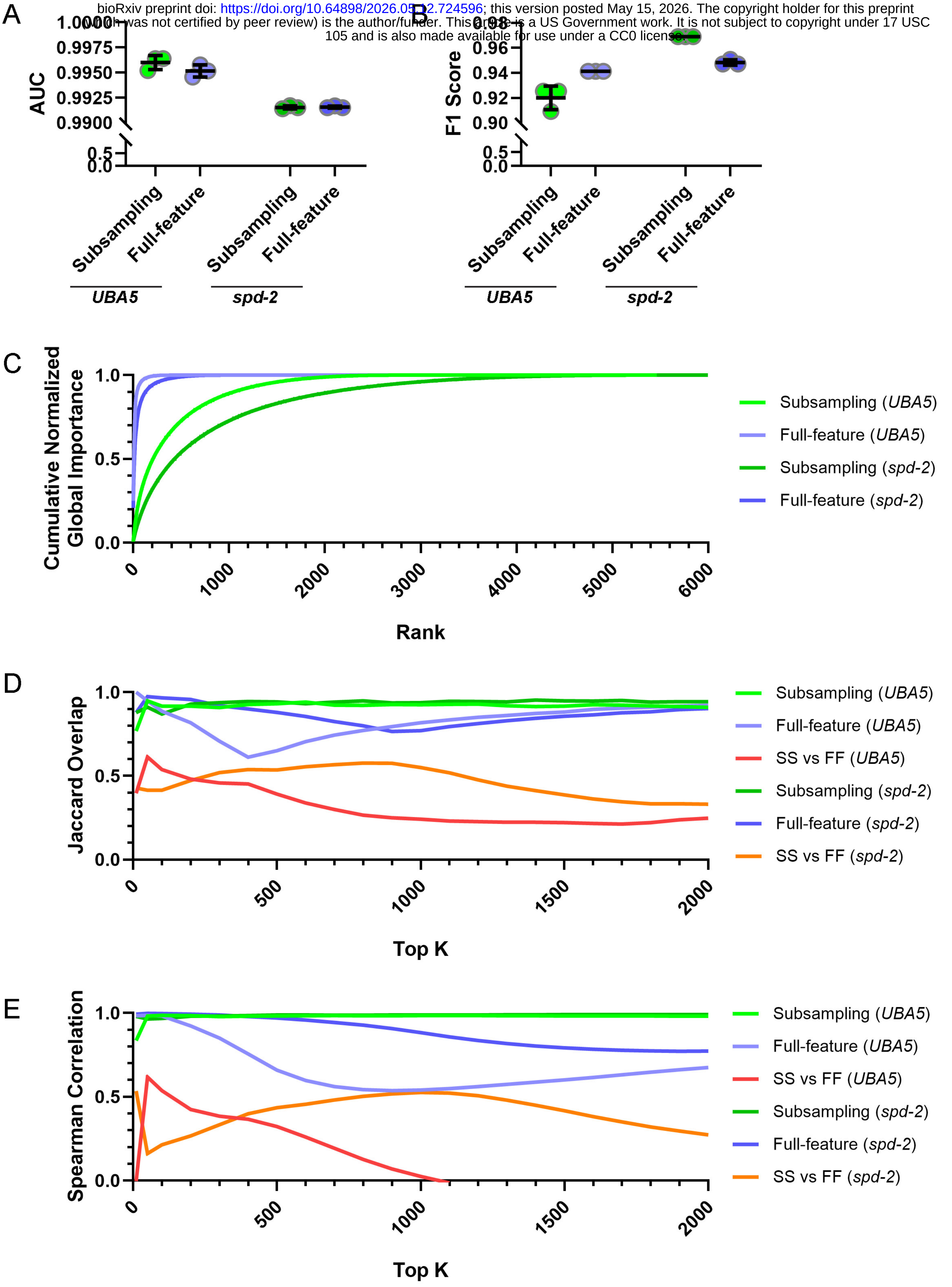

Figure S7 - Subsampling vs Full-feature XGBoost Comparison

bioRxiv preprint doi: https://doi.org/10.64898/2026.05.12.724596; this version posted May 15, 2026. The copyright holder for this preprint (which was not certified by peer review) is the author/funder. This article is a US Government work. It is not subject to copyright under 17 USC 105 and is also made available for use under a CC0 license.

Supplemental Figure 7: Comparison of Subsampling and Full-feature XGBoost Feature-Importance Strategies

Comparison of the subsampling XGBoost and full-feature XGBoost approaches on the *UBA5* and *spd-2* datasets. Classification performance across three independent runs is shown as AUC (A) and F1 score (B). Cumulative normalized global importance (C) shows that full-feature XGBoost concentrates importance in a small number of top-ranked features, whereas the subsampling approach distributes importance more broadly across the ranked list. Jaccard overlap among the top-k globally important features across the three independent runs is shown for the *UBA5* and *spd-2* datasets (D), along with overlap between the subsampling and full-feature approaches within each dataset. Spearman rank correlation on the union of the top-k feature sets is shown for the same comparisons (E). In D and E, subsampling is shown in green, full-feature XGBoost in purple, and between-method comparisons in red/orange, with lighter colors indicating the *UBA5* dataset and darker colors indicating the *spd-2* dataset. SS, subsampling XGBoost; FF, full-feature XGBoost.

bioRxiv preprint doi: https://doi.org/10.64898/2026.05.12.724596; this version posted May 15, 2026. The copyright holder for this preprint (which was not certified by peer review) is the author/funder. This article is a US Government work. It is not subject to copyright under 17 USC 105 and is also made available for use under a CC0 license.

Additional Supplemental Material

Supplemental Methods File

Supplemental Tables 1-21

Video File 1: Examples of the 10 Behavior labels, shown as Floor, Ceiling, and Wall-Side Orientations.

Video File 2: Example inference of *Drosophila* Behavior and Orientation labels.

bioRxiv preprint doi: https://doi.org/10.64898/2026.05.12.724596; this version posted May 15, 2026. The copyright holder for this preprint (which was not certified by peer review) is the author/funder. This article is a US Government work. It is not subject to copyright under 17 USC 105 and is also made available for use under a CC0 license.

Supplemental Methods

Data Pre-processing

Each cropped movie was processed by DeepLabCut, converting the movie into a spreadsheet where each row represents a movie frame and each column represents an x, y, or likelihood value for a given body part. Raw DLC files were further pre-processed to prepare for neural network input. First, centroid (C_x and C_y) position was calculated using the Front (F) and Back (B) keypoints:

$$C_x = \frac{F_x + B_x}{2}$$

$$C_y = \frac{F_y + B_y}{2}$$

Next, body angle (θ_{degrees}) was calculated using the F and B keypoints:

$$\Delta_x = F_x - B_x$$

$$\Delta_y = F_y - B_y$$

$$\theta_{\text{radians}} = atan2(\Delta_y, \Delta_x) * \frac{180}{\pi}$$

$$\theta_{\text{degrees}} = (\theta_{\text{radians}} + 90)(mod\ 360)$$

We performed a series of corrections on the raw x, y keypoints to separate the overall position and angle of the fly from the relationships between body parts. First, we subtracted the centroid coordinates C_x and C_y from all raw keypoints P_x and P_y, pinning the centroid at coordinate (0, 0):

$$P'_x = P_x - C_x$$

bioRxiv preprint doi: https://doi.org/10.64898/2026.05.12.724596; this version posted May 15, 2026. The copyright holder for this preprint (which was not certified by peer review) is the author/funder. This article is a US Government work. It is not subject to copyright under 17 USC 105 and is also made available for use under a CC0 license.

$$P_y' = P_y - C_y$$

Second, we rotated all parts around the center of the plane based on the current angle such that the fly always points North:

$$\theta_{radians} = -(\theta_{degrees} * \frac{\pi}{180})$$

$$P_x'' = P_x' \cos(\theta_{\text{radians}}) - P_y' \sin(\theta_{\text{radians}})$$

$$P_y'' = P_x' \sin(\theta_{\text{radians}}) - P_y' \cos(\theta_{\text{radians}})$$

We engineered multiple additional features to include as input data. Overall animal movement features and part-specific movement features were calculated based on the average change from the previous frame to current frame and current frame to next frame, using raw centroid position or corrected part-specific position, respectively. First, a 2D movement vector (M_t) was calculated for each frame t using the centroid positions (C) from the preceding (t – 1) and subsequent (t + 1) frames:

$$M_{x,t} = \frac{C_{x,t+1} - C_{x,t-1}}{2}$$

$$M_{y,t} = \frac{C_{y,t+1} - C_{y,t-1}}{2}$$

$$M_t = (M_{x,t}, M_{y,t})$$

This instantaneous movement vector was then used to create three separate features. Velocity (magnitude; $v_{mag,t}$) represents the overall speed of the animal or part, regardless of direction:

$$v_{mag,inst,t} = |M_t| = \sqrt{M_{x,t}^2 + M_{y,t}^2}$$

bioRxiv preprint doi: https://doi.org/10.64898/2026.05.12.724596; this version posted May 15, 2026. The copyright holder for this preprint (which was not certified by peer review) is the author/funder. This article is a US Government work. It is not subject to copyright under 17 USC 105 and is also made available for use under a CC0 license.

Forward velocity ($v_{fwd,t}$) and strafe velocity ($v_{strafe,t}$) represent the component of the animal's movement that is parallel and perpendicular to the body axis, respectively, and were calculated for the whole body movement. First, the body axis vector A_t is defined using the Back (B) and Front (F) keypoints:

$$A_t = F_t - B_t = (F_{x,t} - B_{x,t}, F_{y,t} - B_{y,t})$$

Additionally, we defined a pair of perpendicular axes using Left/Right (A_{LR}) and Top/Bottom (A_{TB}) keypoints. For each frame, the perpendicular axis used is based on which one has the higher summed keypoint likelihood:

$$A_{LR,t} = EyeRight_t - EyeLeft_t$$

$$A_{TB,t} = ThoraxBottom_t - ThoraxTop_t$$

$$S_t = \begin{cases} A_{LR,t} \; if \; C_{LR} \geq C_{TB} \\ A_{TB,t} \; if \; C_{LR} < C_{TB} \end{cases}$$

These vectors are normalized to create unit vectors ($\hat{A}_t$ and $\hat{S}_t$) that point in the forward and strafe directions, respectively:

$$\hat{A}_t = \frac{A_t}{|A_t|}$$

$$\hat{S}_t = \frac{S_t}{|S_t|}$$

Finally the forward velocity is calculated by projecting the M_t onto the normalized vectors $\hat{A}_t$ and $\hat{S}_t$:

$$v_{fwd,inst,t} = M_t * \hat{A}_t$$

$$v_{strafe,inst,t} = M_t * \hat{S}_t$$

bioRxiv preprint doi: https://doi.org/10.64898/2026.05.12.724596; this version posted May 15, 2026. The copyright holder for this preprint (which was not certified by peer review) is the author/funder. This article is a US Government work. It is not subject to copyright under 17 USC 105 and is also made available for use under a CC0 license.

Acceleration was calculated based on the change in $v_{inst,t}$ (using $v_{mag,t}$, $v_{fwd,t}$, and $v_{strafe,t}$ to calculate $a_{mag,t}$, $a_{fwd,t}$, and $a_{strafe,t}$, respectively) using the formula:

$$a_{inst,t} = \frac{(v_{inst,t} - v_{inst,t-1}) + (v_{inst,t+1} - v_{inst,t})}{2}$$

Jerk was similarly calculated for the animal centroid based on the change in $a_{inst,t}$ (using $a_{mag,t}$, $a_{fwd,t}$, and $a_{strafe,t}$ to calculate $j_{mag,t}$, $j_{fwd,t}$, and $j_{strafe,t}$,, respectively) using the formula:

$$j_{inst,t} = \frac{(a_{inst,t} - a_{inst,t-1}) + (a_{inst,t+1} - a_{inst,t})}{2}$$

Next, angular velocity was calculated as the change in angle between a “moving” keypoint as it rotates around a “base” keypoint. Whole body angular velocity was calculated using the Front (moving) and Back (base) keypoints, and part-specific angular velocities were calculated based on pairs listed in Supplemental Table 3, using the formula:

$$\Delta x_t = P_{moving,x,t} - P_{base,x,t}$$

$$\Delta y_t = P_{moving,y,t} - P_{base,y,t}$$

$$\Delta\theta_{pair,t} = atan2(\Delta y_t, \Delta x_t) * \frac{180}{\pi}$$

$$\Delta\theta_{prev} = \theta_{pair,t} - \theta_{pair,t-1}$$

$$\Delta\theta_{next} = \theta_{pair,t+1} - \theta_{pair,t}$$

Both $\Delta\theta$ values are normalized to the range [-180, 180], and used to calculate part-specific angular velocity $\omega_{pair,t}$:

$$\omega_{pair,inst,t} = \frac{\Delta\theta_{prev,norm} + \Delta\theta_{next,norm}}{2}$$

bioRxiv preprint doi: https://doi.org/10.64898/2026.05.12.724596; this version posted May 15, 2026. The copyright holder for this preprint (which was not certified by peer review) is the author/funder. This article is a US Government work. It is not subject to copyright under 17 USC 105 and is also made available for use under a CC0 license.

Angular acceleration ($\alpha_{inst,t}$) was calculated based on the change in $\omega_{pair,t}$ using the formula:

$$\alpha_{inst,t} = \frac{\omega_{inst,t+1} - \omega_{inst,t-1}}{2}$$

Angular jerk ($j_{ang,pair,t}$) was calculated based on the change in $\alpha_{inst,t}$ using the formula:

$$j_{ang,inst,t} = \frac{\alpha_{inst,t+1} - \alpha_{inst,t-1}}{2}$$

Path curvature was calculated as the ratio of the animal's angular velocity to its forward velocity:

$$k_{inst,t} = \frac{\omega_{inst,t}}{v_{mag,inst,t} + \epsilon}$$

Rolling averages for all velocity, acceleration, jerk, angular velocity, angular acceleration, angular jerk, and curvature were calculated by averaging the instantaneous values from five or nine consecutive frames, centered on the current frame. We also calculated standard deviation features based on the instantaneous values across five- and nine-frame windows for all velocity and angular velocity features.

To help the model learn a fly's orientation, we calculated a series of log likelihood ratios λ_{ratio} using the likelihood values of opposite body part pairs (L_1 and L_2; Supplemental Table 4), under the assumption that visible points will have high likelihood and hidden points will have low likelihood:

$$\lambda_{ratio} = \ln\left(\frac{L_1 + 10^{-6}}{L_2 + 10^{-6}}\right)$$

We calculated static length features as the distance between selected pairs of keypoints (Supplemental Table 5):

$$L_{AB} = \sqrt{(A_x - B_x)^2 + \left(A_y - B_y\right)^2}$$

We calculated static angle features (ψ) as the angle formed by three keypoints (a base B and two limbs A and C; Supplemental Table 6):

bioRxiv preprint doi: https://doi.org/10.64898/2026.05.12.724596; this version posted May 15, 2026. The copyright holder for this preprint (which was not certified by peer review) is the author/funder. This article is a US Government work. It is not subject to copyright under 17 USC 105 and is also made available for use under a CC0 license.

$$\overrightarrow{BA} = (A_x - B_x, A_y - B_y)$$

$$\overrightarrow{BC} = (C_x - B_x, C_y - B_y)$$

$$\psi_{triplet} = \arccos\left(\frac{\overrightarrow{BA} * \overrightarrow{BC}}{|\overrightarrow{BA}| * |\overrightarrow{BC}|}\right) * \frac{180}{\pi}$$

Finally, we calculated head orientation angle features which measure the orientation of the head, based on either the Left and Right eyes (R and L) or the Ocelli and Mouthparts (O and M) relative to the main body F-B axis. When the fly is standing in neutral position, these features should equal 90°; as the fly looks to the right or downwards, the features approach 0°, and as the fly looks to the left or upwards the features approach 180°:

$$\overrightarrow{FB} = (B_x - F_x, B_y - F_y)$$

$$\overrightarrow{LR} = (R_x - L_x, R_y - L_y)$$

$$\overrightarrow{MO} = (O_x - M_x, O_y - M_y)$$

$$|\psi_{head1}| = \arccos\left(\frac{\overrightarrow{FB} * \overrightarrow{LR}}{|\overrightarrow{FB}| * |\overrightarrow{LR}|}\right) * \frac{180}{\pi}$$

$$|\psi_{head2}| = \arccos\left(\frac{\overrightarrow{FB} * \overrightarrow{MO}}{|\overrightarrow{FB}| * |\overrightarrow{MO}|}\right) * \frac{180}{\pi}$$

Area features were calculated as the area enclosed by specific sets of keypoints (Supplemental Table 7).

bioRxiv preprint doi: https://doi.org/10.64898/2026.05.12.724596; this version posted May 15, 2026. The copyright holder for this preprint (which was not certified by peer review) is the author/funder. This article is a US Government work. It is not subject to copyright under 17 USC 105 and is also made available for use under a CC0 license.

Asymmetry indexes were calculated to capture the left-right or top-bottom asymmetry of specific keypoint pairs (Supplemental Table 8) using the corrected keypoints:

$$Asym_{pair} = ||P_1| - |P_2||$$

Altogether, the full pre-processed data contains 820 feature columns. Each column of data is z-score normalized across the entire dataset, such that mean is 0 and standard deviation is +/- 1. To optimize loading speed, the final normalized data files are saved in binary HDF5 format with 32-bit floating point precision.

Post-inference Feature Engineering

After generating Behavior and Orientation (BxO) predictions for an experimental dataset, we performed extensive post-inference analysis to characterize behavioral differences between groups. The first step was to extract a high-dimensional feature matrix for each individual animal using a combination of bout and keypoint-based data. First, we extracted bouts of contiguous predicted labels: Behavior-only bouts, Orientation-only bouts, and BxO bouts. We then calculated high-level bout summary features. Bout frequency was calculated as the number of bouts divided by the total video time in seconds:

$$bout_{frequency} = \frac{n_{bouts}}{Time_{total}}$$

Mean bout duration was calculated:

$$\mu_L = \frac{\sum bout_{length}}{n_{bouts}}$$

$$bout\_duration_{mean} = \frac{\mu_L}{40\ fps}$$

Bout duration coefficient of variation was calculated:

$$\sigma_L = \sqrt{\frac{\sum(bout_{length} - \mu_L)^2}{n_{bouts}}}$$

bioRxiv preprint doi: https://doi.org/10.64898/2026.05.12.724596; this version posted May 15, 2026. The copyright holder for this preprint (which was not certified by peer review) is the author/funder. This article is a US Government work. It is not subject to copyright under 17 USC 105 and is also made available for use under a CC0 license.

$$bout\_duration\_CV = \frac{\sigma_L}{\mu_L}$$

Initiation probability estimates the probability of starting a new bout when the animal is not already engaged in that bout:

$$F_{available} = F_{total} - bout_{total\ frames}$$

$$initiation\_prob = \frac{n_{bouts}}{F_{available}}$$

Inter-bout interval was calculated based on the time between the end of the previous bout and the start of the next bout of the same type.

$$IBI_{frames} = [start_{bout\ 2} - end_{bout\ 1}, start_{bout\ 3} - end_{bout\ 2}, \ldots]$$

The mean and standard deviation of these intervals were then calculated and converted to seconds.

Next, time budgets were calculated for each bout type representing the total proportion of recorded time that the individual spent performing a given bout type:

$$Time\ Budget = \frac{Total\ frames\ per\ bout\ type}{Total\ recorded\ frames}$$

We also calculated conditional time budgets. $P(Behavior|Orient)$ represents the proportion of time the individual spent performing a given Behavior relative to the total time spent in a specific Orientation. Conversely, $P(Orient|Behavior)$ represents the proportion of time the individual spent in a given Orientation relative to the total time spent performing a specific Behavior:

$$P(Behavior|Orient) = \frac{Total\ frames\ for\ given\ Behavior + Orient}{Total\ frames\ for\ given\ Orient}$$

$$P(Orient|Behavior) = \frac{Total\ frames\ for\ given\ Orient + Behavior}{Total\ frames\ for\ given\ Behavior}$$

Standardized body-size features were calculated for each animal using Orientation-specific keypoint distances and body-area measurements (Supplemental Table 12). For each measurement, frames were first restricted to the required Orientation label and then searched using a Behavior hierarchy (Standing,

bioRxiv preprint doi: https://doi.org/10.64898/2026.05.12.724596; this version posted May 15, 2026. The copyright holder for this preprint (which was not certified by peer review) is the author/funder. This article is a US Government work. It is not subject to copyright under 17 USC 105 and is also made available for use under a CC0 license.

Walking, Groom-FrontLegs, Groom-Head, Groom-SideLeg, Groom-Wings, Groom-Abdomen, Groom-BackLegs), stopping at the first Behavior yielding valid frames. From up to 100 valid frames per recording, we calculated the mean and standard deviation of each measurement. Measurement definitions included top-, bottom-, and side-view body length, head, thorax, and abdomen dimensions, left and right wing length, and body area. Recording-level values were averaged across recordings for each animal. Averaged body-size features were calculated by combining top- and bottom-view measurements, with side-view measurements used as fallbacks when top- and bottom-view data were unavailable. Normalized size features were obtained by dividing linear measurements, or the square root of body area, by the averaged thorax width. See Supplemental Table 12 for all measurements.

Postural features were calculated for each animal to characterize standing posture and body-part asymmetry. Postural features were extracted from Standing frames only, and were aggregated within each animal and Orientation by computing the mean and standard deviation across valid frames. For Floor- and Ceiling-oriented Standing frames, we retained only frames in which all required leg-tip and body-axis keypoints had likelihood > 0.1. We then calculated stance area as the polygonal area enclosed by the six leg tips, and calculated front-, mid-, and back-leg extension as the mean distance of each left-right leg pair from the body centroid:

$$d_{left} = \sqrt{(x_{tarsus\ left} - x_{centroid})^2 + (y_{tarsus\ left} - y_{centroid})^2}$$

$$d_{right} = \sqrt{(x_{tarsus\ right} - x_{centroid})^2 + (y_{tarsus\ right} - y_{centroid})^2}$$

$$extension\_raw\_[Pair]\_avg = \frac{d_{left} + d_{right}}{2}$$

For Floor-oriented Standing frames, we also calculated left-right asymmetry magnitudes for the eyes, thorax, abdomen, wings, and each leg pair using the precomputed x- and y-asymmetry indices, and summarized these as an overall left-right asymmetry score. For Standing frames with Wall-Side Orientation prediction ≥ 0.75, top-bottom asymmetry magnitudes for the head, thorax, and abdomen were calculated and summarized as overall top-bottom asymmetry scores.

$$asymmetry\_magnitude\_[Pair]\_[Type] = \sqrt{(asym\_x)^2 + (asym\_y)^2}$$

bioRxiv preprint doi: https://doi.org/10.64898/2026.05.12.724596; this version posted May 15, 2026. The copyright holder for this preprint (which was not certified by peer review) is the author/funder. This article is a US Government work. It is not subject to copyright under 17 USC 105 and is also made available for use under a CC0 license.

Two overall postural asymmetry features (overall_asymmetry_LR and overall_asymmetry_TB) were calculated as the mean of all LR or TB asymmetry magnitudes.

Wing-spread and left-right leg-pair spread angles were summarized within selected Standing, Walking, and Groom-Wings contexts by calculating the mean and 95th percentile of each angle feature across valid frames.

Kinematic features were calculated from keypoint-derived instantaneous movement signals within valid BxO contexts. Most Behaviors were separated into Floor, Wall-Side, and Ceiling Orientation contexts, while Jump and Scramble retained as collapsed orientation-independent contexts. Wall-Up and Wall-Down were excluded from kinematic features. Instantaneous kinematic signals included body translation, acceleration, and jerk; angular velocity, acceleration, and jerk; walking curvature; head movement; and front-, middle-, and back-leg translational and angular movement. Head movement was derived from five head keypoints by dropping the lowest-likelihood head keypoint on each frame and averaging the remaining signals.

Kinematic features were summarized as bout-based features by first summarizing each bout individually and then aggregating those summaries across bouts of the same type within each animal. Non-walking bouts were summarized over the full bout, whereas walking bouts were summarized using a trimmed core window together with separate start and end windows of five frames each. For each bout, we calculated the mean, standard deviation, median, Pearson's non-parametric skewness, and robust minimum and maximum values defined as the mean of the bottom and top 5% of values. Only features derived from at least three valid bouts were retained. We also calculated a global locomotor summary feature by averaging whole-body speed across all frames for each animal.

To calculate transition probabilities for Behavior labels and combined BxO labels, we identified successive transitions in which one label was followed by a different label, such that each transition had a "From" and "To" state. If $N_{From>To}$ is the number of transitions from the From state to the To state, and $N_{Total\ From}$ is the total number of outgoing transitions from that From state, then transition probability was calculated as:

$$P(To|From) = \frac{N_{From>To}}{N_{Total\ From}}$$

When calculating transitions for the BxO combined labels, we collapsed all Jump labels into a single category due to their rarity, and collapsed all Scramble labels into a single category due to the

bioRxiv preprint doi: https://doi.org/10.64898/2026.05.12.724596; this version posted May 15, 2026. The copyright holder for this preprint (which was not certified by peer review) is the author/funder. This article is a US Government work. It is not subject to copyright under 17 USC 105 and is also made available for use under a CC0 license.

rapid transitions between Orientations. Transition features supported by fewer than six outgoing transitions from the source state were masked downstream.

Spatial and directional features were calculated from the centroid position and body orientation of the animal. First, we converted the centroid to a rotationally-invariant polar coordinate system, where (299.5, 299.5) is considered the chamber center:

$$r = \sqrt{(x_{centroid} - x_{chamber})^2 + (y_{centroid} - y_{chamber})^2}$$

We then used the chamber radius to calculate a normalized radius r_{norm}, where the center is 0 and the wall is 1:

$$r_{norm} = \frac{r}{R_{arena}}$$

For each behavior or BxO combination, we collected all r_{norm} values and calculated radial-position statistics including mean, median and standard deviation.

We calculated relative angle, where 0° points directly away from the chamber center and 180° is pointing directly towards the chamber center, by subtracting the positional angle θ_{pos} from its actual body angle α_{fly}:

$$\theta_{pos} = atan2(y - \mu_y, x - \mu_x)$$

$$relative\_angle = \alpha_{fly} - \theta_{pos}$$

We calculated center and periphery occupancy fractions by classifying normalized radial positions as center ($r_{norm} \leq 0.4$) or periphery ($r_{norm} \geq 0.7$) and computing the proportion of frames in each region within selected behavioral contexts.

We also quantified directional orientation relative to the chamber center by calculating the framewise angle between the body axis and the radial position vector. After excluding frames near the chamber center ($r_{norm} < 0.1$), we summarized the mean and standard deviation of the absolute relative angle within each behavioral context.
